# Supplementary material for: Completing bacterial genome assemblies with multiplex MinION sequencing
Source: Microb Genom. 2017 Sep 14;3(10):e000132. doi: 10.1099/mgen.0.000132 (PMC5695209; doi:10.1099/mgen.0.000132)
Supplement: Supplementary File 1 [file mgen-3-132-s001.pdf]

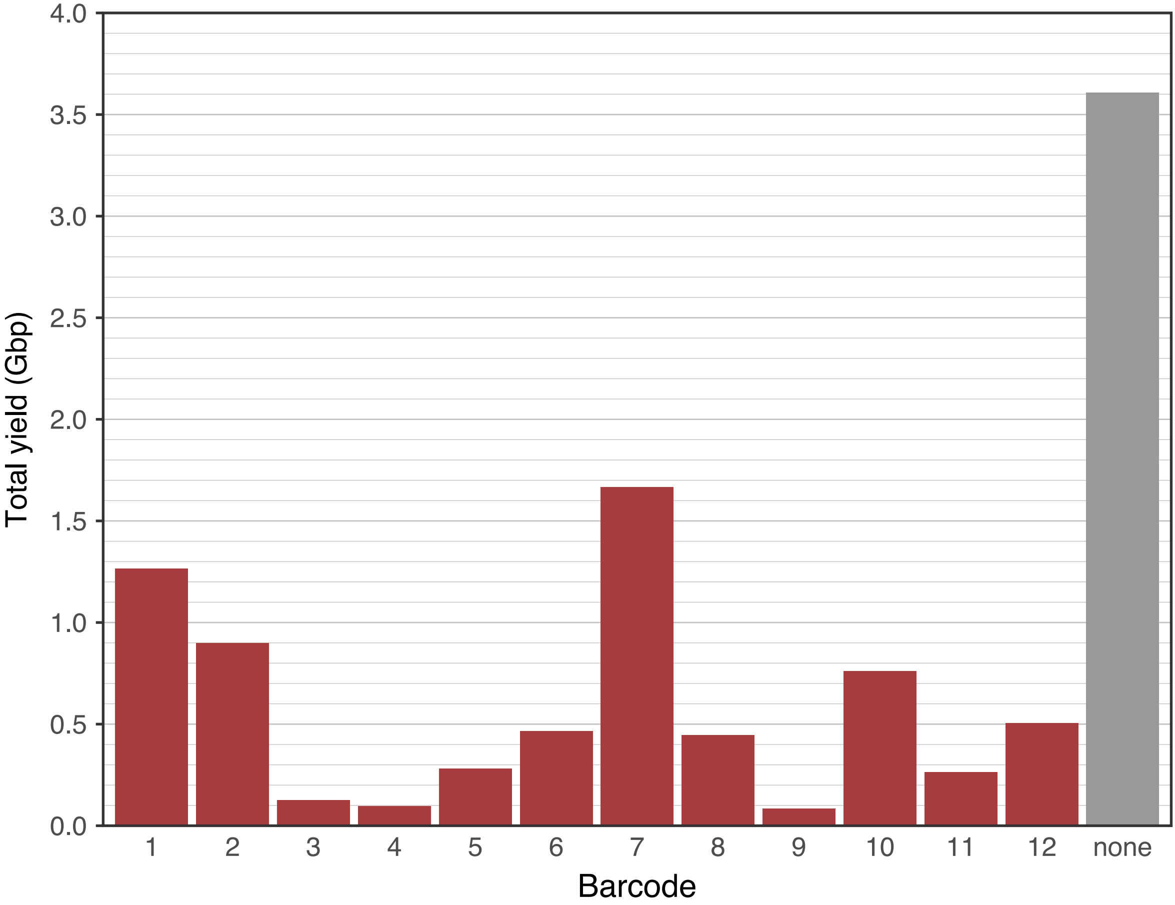

4.0
3.5
3.0
2.5
2.0
1.5
1.0
0.5
0.0
Total yield (Gbp)
1
2
3
4
5
6
7
8
9
10
11
12
none
Barcode

Sample	ONT reads	Assembly graph	Estimated error rate: Before Nanopolish	Estimated error rate: After Nanopolish
INF042	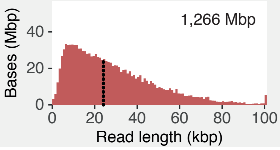	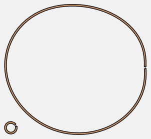	0.807%	0.349%
INF059	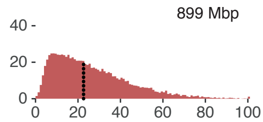	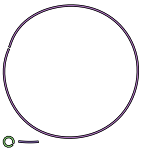	0.947%	0.441%
INF163	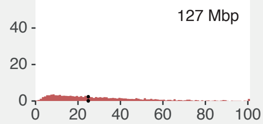	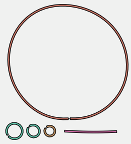	1.500%	0.878%
INF164	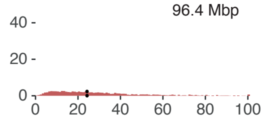	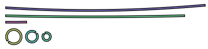	1.690%	1.094%
INF249	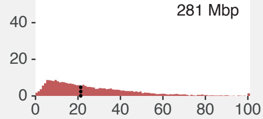	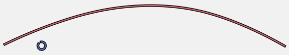	1.229%	0.646%
INF322	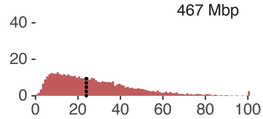	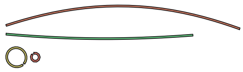	1.155%	0.601%
KSB1_7G	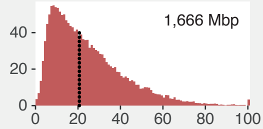	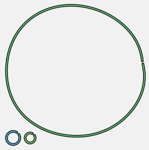	0.994%	0.574%
KSB1_7J	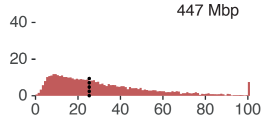		1.131%	0.554%
KSB1_8D	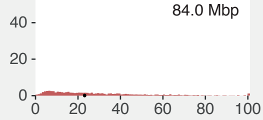	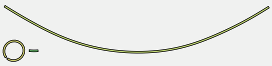	1.884%	1.232%
KSB1_9A	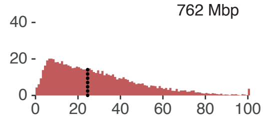	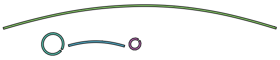	0.896%	0.356%
KSB1_9D	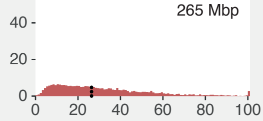	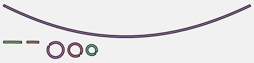	1.262%	0.680%
KSB2_1B	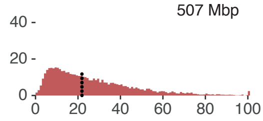	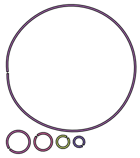	1.192%	0.635%

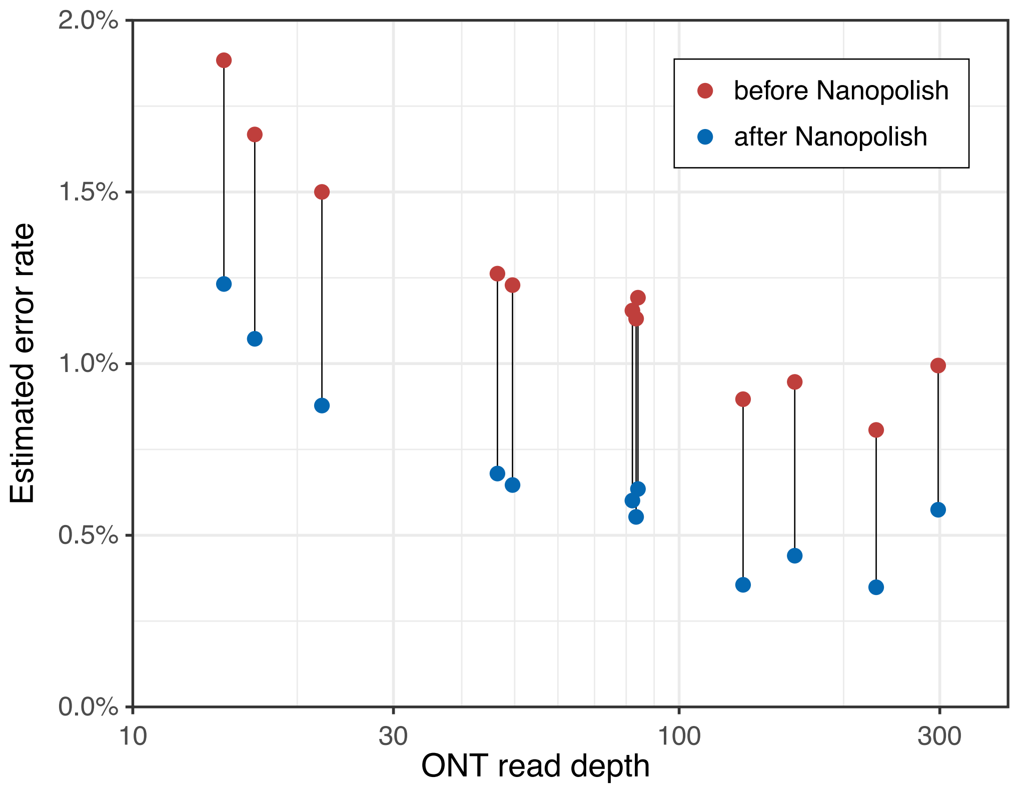

2.0%
1.5%
1.0%
0.5%
0.0%
Estimated error rate
10
30
100
300
ONT read depth
before Nanopolish
after Nanopolish

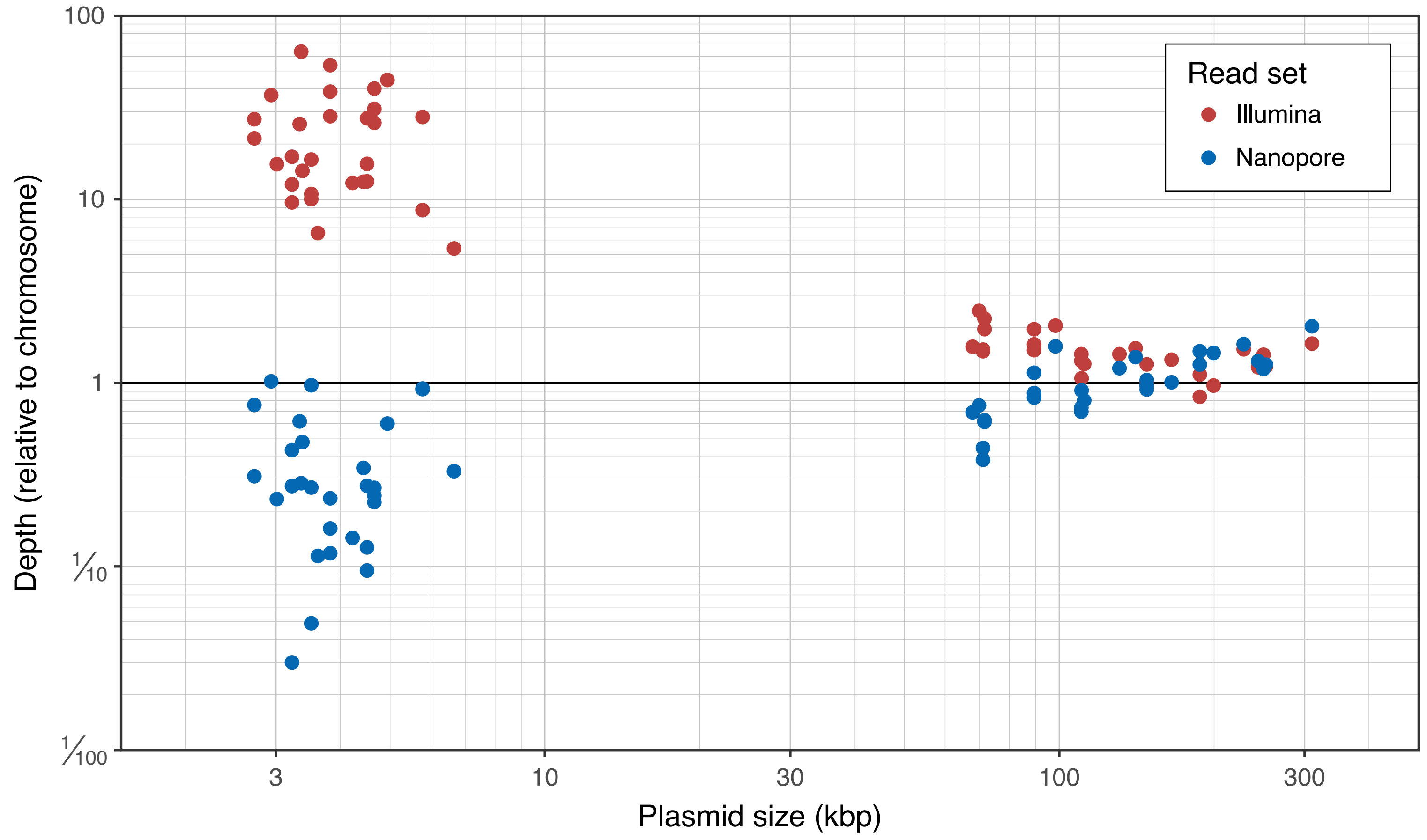

Read set
Illumina
Nanopore
Depth (relative to chromosome)
100
10
1
1⁄10
1⁄100
Plasmid size (kbp)
3
10
30
100
300
